# Robust Circularly Polarized Luminescence via Quasi-Bound States in the Continuum in Intrinsic Chiral Silicon Metasurfaces

**DOI:** 10.1021/acsphotonics.5c01966

**Published:** 2025-10-27

**Authors:** Xiao-Ke Zhu, Yu-Chen Wei, Jose L. Pura, Matthijs Berghuis, Minpeng Liang, Beatriz Castillo López de Larrinzar, Shunsuke Murai, Antonio García-Martín, José A. Sánchez-Gil, Sailing He, Jaime Gómez Rivas

**Affiliations:** † Department of Applied Physics and Science Education, 3169 Eindhoven University of Technology, Eindhoven 5600MB, The Netherlands; ‡ Centre for Optical and Electromagnetic Research, National Engineering Research Center for Optical Instruments, Zhejiang University, Hangzhou 310058, China; § Instituto de Estructura de la Materia (IEM-CSIC), 16379 Consejo Superior de Investigaciones Científicas, Serrano 121, Madrid 28006, Spain; ∥ Gds-Optronlab, Fisica de la Materia Condensada, Universidad de Valladolid, Paseo de Belén 19, Valladolid 47011, Spain; ⊥ Instituto de Micro Y Nanotecnología IMN, CSIC, CEI UAM+CSIC, Isaac Newton 8, Tres Cantos, Madrid E-28760, Spain; # Department of Physics and Electronics, Graduate School of Engineering, 12936 Osaka Metropolitan University, Osaka 599-8531, Japan

**Keywords:** surface lattice resonance, mode decomposition, chiral light-matter interaction, lateral field confinement, emitter layer thickness

## Abstract

We demonstrate circularly polarized photoluminescence emission, with dissymmetry factors *g*_PL_ over 0.1, from achiral organic dye molecules by leveraging quasi-bound states in the continuum (quasi-BICs) and surface lattice resonances (SLRs) in intrinsic silicon chiral metasurfaces. We find that the *g*_PL_ associated with the quasi-BIC mode remains robust against variations in the emission angle and dye thickness due to its strong lateral field confinement. In contrast, the *g*_PL_ of the SLR mode exhibits sign inversion depending on the emission energy and the dye layer thickness. The experimental results are supported by mode decomposition analysis, helicity density analysis, and the near-field spatial distribution of the electric field. These findings illustrate the relevance of the emitter’s layer thickness in optimizing the emission of circularly polarized light. They also elaborate on the robustness of chiral quasi-BICs by comparing the *g*_PL_ of SLRs and quasi-BICs, offering insights into chiral light-matter interactions and advancing the design of circularly polarized light-emitting devices.

## Graphical abstract

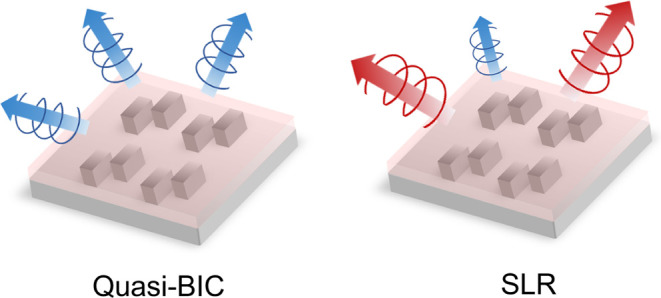

## Introduction

Objects that cannot be superimposed on their mirror images by translations and rotations exhibit intrinsic chirality, which can be observed not only in matter but also in the polarization states of light.^1,2^ Circularly polarized light, a manifestation of chirality in light, offers additional degrees of freedom for encoding information compared to linearly polarized light. This advantage makes chiral luminophores exhibiting circularly polarized luminescence (CPL) particularly interesting for advanced technologies, including 3D displays and augmented reality, optical data storage, optical communication, metrology, imaging, microscopy, and medical diagnostics.^3–11^ The degree of circular polarization in light can be quantified by the photoluminescence dissymmetry factor, *g*_PL_, defined as *g*_PL_ = 2­(*I*_LCP_ – *I*_RCP_)/(*I*_LCP_ + *I*_RCP_), where *I*_LCP_ and *I*_RCP_ represent the intensities of left-handed and right-handed CPL, respectively.^12,13^ Traditionally, sources of CPL are fabricated using bulky optical elements, such as polarizers and quarter-wave plates,^14,15^ which are unsuitable for micro- or nanoscale device platforms. Direct generation of chiral emission from materials offers a pathway toward compact device development. Materials such as chiral perovskites, small chiral organic dyes, and metal complexes have shown promise for generating circularly polarized emission.^16–18^ However, these materials often face challenges, including low *g*_PL_ or low internal/external quantum efficiencies. Nowadays, achieving high *g*_PL_ and quantum efficiencies at room temperature remains challenging.^16–18^

To address this challenge, various types of chiral nanostructures can be applied to enhance *g*_PL_, including plasmonic, excitonic, self-assembled, and photonic systems.^19–24^ Among these, metasurfaces have emerged as a versatile platform for chiro-optical studies, offering unprecedented design flexibility compared to natural materials.^9^ The highly enhanced chiral light-matter interactions in metasurfaces are crucial for applications such as enantiomer selection, chiral molecular sensing, and chiral quantum optics.^9,25–27^ Periodic arrays of nanoparticles in metasurfaces can induce a range of optical phenomena through the radiative coupling of local dipoles and multipoles in each particle. For example, surface lattice resonances (SLRs) arise from the hybridization between localized resonances and Rayleigh anomalies,^28–30^ which have been utilized to achieve significant chiral photoluminescence in quantum dots, perovskites, and molecular dyes.^31–38^ Additionally, if the particle arrays restrict the available radiation channels, these modes remain localized to the structure even though they coexist with the continuum of radiative modes, giving rise to bound states in the continuum (BICs) with theoretically infinite temporal field confinement.^39,40^ In addition, by the introduction of in-plane asymmetry, BICs turn into quasi-BICs with finite but still large field confinements. These optical modes enable significant chiral photoluminescence (CPL) as well as applications in chiral sensing.^41–49^

In this work, we investigate the CPL from an achiral organic dye enabled by SLRs and quasi-BICs in intrinsic chiral silicon metasurfaces. Leveraging these modes, we achieve *g*_PL_ exceeding 0.1. The quasi-BIC mode exhibits robust CPL with a consistent *g*_PL_ of 0.1, remaining stable across variations in dye thickness and emission angle. In contrast, the SLR mode demonstrates a higher *g*_PL_ of 0.17. Notably, the sign of *g*_PL_ within the same SLR mode can be inverted by altering the dye thickness or the emission angle. These behaviors are elucidated through analysis of the local electric field distribution, helicity density, and modal decomposition analysis. Our findings highlight the comparative robustness of CPL between SLR and quasi-BIC modes in intrinsic chiral metasurfaces, paving the way for the development of advanced chiral luminescent devices with robust emission.

## Sample Description and Photoluminescent Enhancements

The metasurfaces consist of a matrix of polycrystalline silicon nanorod dimers with height *L*_*z*_ = 90 nm, width *L*_*x*_ = 50 nm, and length *L*_*y*_ = 130 nm on a glass substrate, placed in a square lattice with a unit cell of 340 × 340 nm^2^ (see Figure 1a for a schematic illustration). For the configuration with maximum symmetry (Figure 1b), the distance between the two rods along the *x*-axis (*D*_*x*_ = 115 nm) is large enough to analyze the two rods within the dipolar approximation. To manipulate the chiro-optical activity, we study the intrinsic dissymmetry arising from the displacements of one nanorod along the *y*-axis (*D*_*y*_ = ±96 nm) (Figure 1c,d). Note that the unit cell with the opposite sign of *D*_*y*_ is an enantiomorph. As a result of symmetry restrictions, we expect the chiro-optical responses from these enantiomorphs to be inverse to each other. The sample area for each sample is 2 × 2 mm^2^. The details of the sample fabrication can be found in the Methods section. Figure 1b–d shows the scanning electron microscopy (SEM) images of the samples, which illustrate the designed structural dimensions.

**Figure 1:**
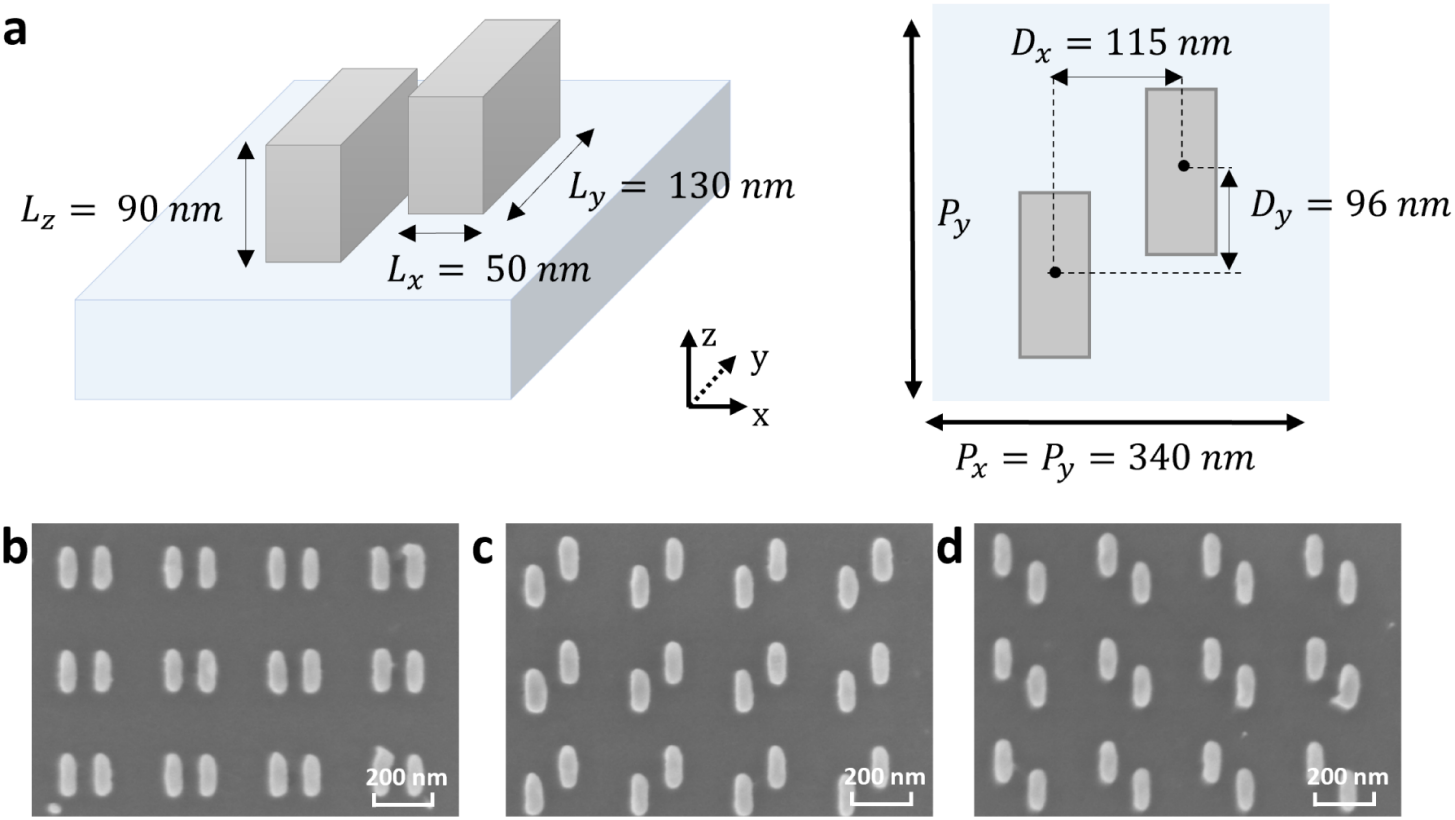
Chiral metasurfaces formed by arrays of silicon nanorod dimers. (a) Scheme of one unit cell of the chiral metasurface composed of two displaced Si nanorods. The structural parameters are indicated. The dotted line of the *y*-axis indicates the direction into the plane. (b–d) SEM images of three metasurfaces with unit cells formed by (b) equal, nonvertically displaced Si nanorods (achiral), (c) positively displaced Si nanorods in the *y*-direction, and (d) negatively displaced Si nanorods in the *y*-direction.

After the fabrication of the metasurfaces, we spin-coated a 220 nm thick layer of 5 wt % perylene dye ([*N*,*N*′-bis (2,6-diisopropylphenyl)-1,7- and -1,6-bis­(2,6-diisopropylphenoxy)­perylene-3,4:9,10-tetracarboximide]) in poly­(methyl methacrylate) (PMMA) on top. The bare perylene dye shows two exciton peaks in both the extinction and photoluminescence (PL) spectra, corresponding to the electronic transition and its first vibronic replica, respectively (see Figure  S1). We used a continuous-wave laser with a wavelength of 532 nm to excite the three samples and measured their angle-dependent PL enhancement (PLE) with a Fourier microscope. The PLE was analyzed as a function of the emission wavelength and the in-plane wavevector (parallel to the surface) along the *x*-axis (*k*_*x*_). The Fourier microscope used for these measurements is schematically shown in Figure  S2. The PLE was obtained from the PL maps of the dye on metasurfaces divided by the PL maps measured for a bare organic layer. To avoid polarized excitation from the laser source, we added an optical diffuser in front of the sample to achieve unpolarized excitation. In Figure 2, the PLE dispersion measurements are shown for the parallel wavevector along the short axis of the nanorods (*x*-axis). The dispersion along the *x*-axis shows two distinct modes. The mode with larger enhancements (PLE ≈ 4–5) and a high quality factor (*Q* ≈ 69) corresponds to a BIC (Figure 2a). The PLE vanishes in the direction normal to the surface (*k_x_* = 0) due to the inversion symmetry of the arrays along this direction. This inversion symmetry is the origin of the symmetry protection, leading to the suppression of far-field emission.^50–52^ For wavevectors other than 0, the inversion symmetry is broken, and radiation leakage is possible, giving rise to the quasi-BIC modes. By contrast, the mode with smaller enhancements (PLE ≈ 1.7), a relatively low quality factor (*Q* ≈ 32), and radiation leakage at normal incidence and higher *k*_*x*_ compared to BIC corresponds to the SLRs (Figure 2b,c). The stronger PLE from the BIC and quasi-BIC is associated with their higher *Q*-factor and field confinement to the surface.^53,54^ Note that exhibiting both SLR and quasi-BIC on the same array offers an effective platform for comparing their photonic characteristics. The PLE measurements with in-plane wavevectors along the *y*-axis are shown in Figure  S3. There is only one SLR mode visible with significant PLE (over a factor of 8) and one low-dispersion band at 2.1 eV with a weak PLE ≈ 1.

**Figure 2:**
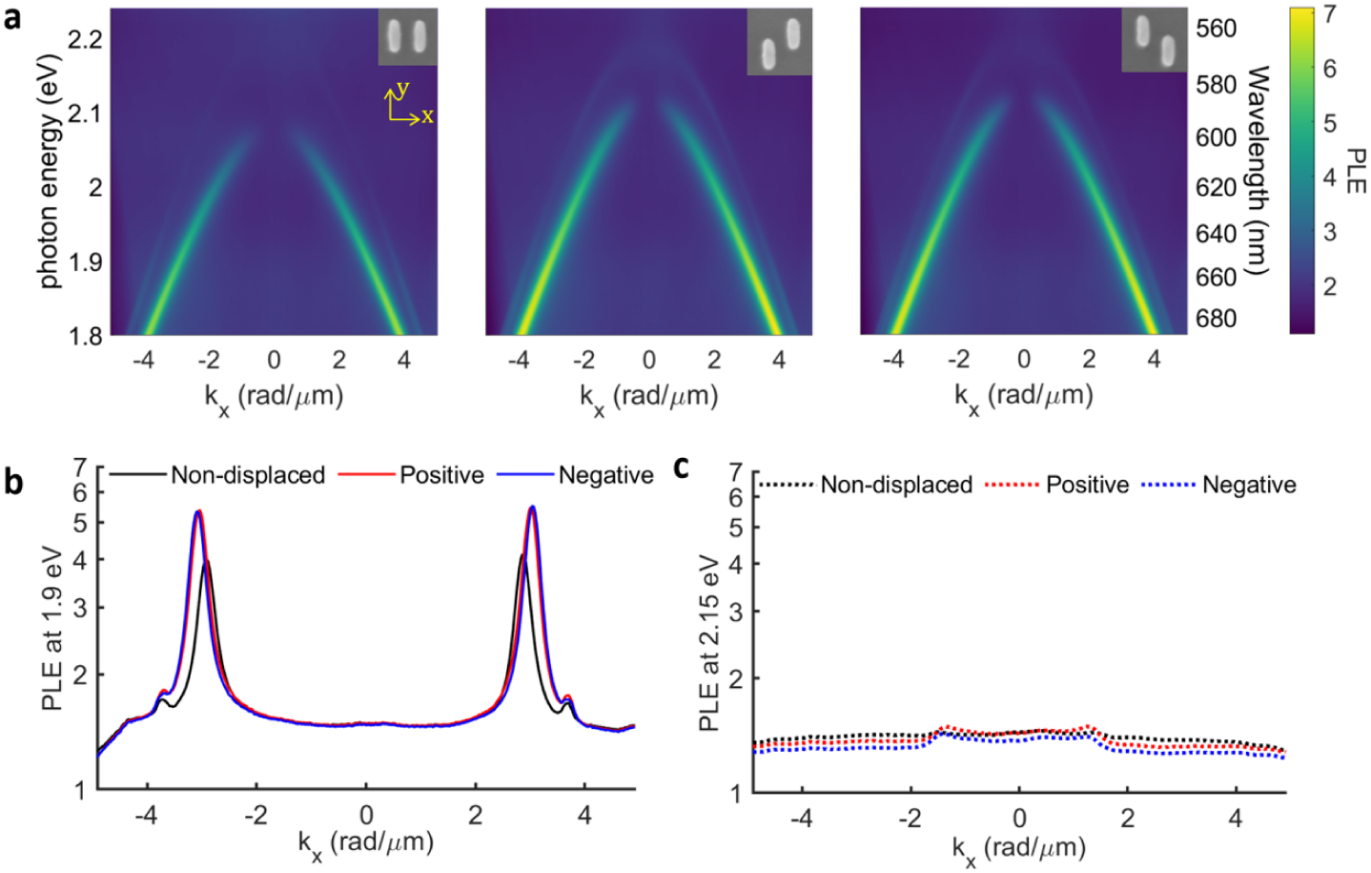
Photoluminescence (PL) analysis for three metasurfaces of nanorod dimers with a spin-coated dye-doped polymer film on top with a thickness of 220 nm. (a) Photoluminescence enhancement (PLE) for metasurfaces with a unit cell formed by two equal, nonvertically displaced Si nanorods (left panel), two positively displaced Si nanorods in the *y*-direction (central panel), and two negatively displaced Si nanorods in the *y*-direction (right panel), measured with a Fourier microscope as a function of the in-plane wave vector *k*_*x*_. The insets show SEM images of the unit cell for each structure. PLE as a function of *k*_*x*_ at (b) 1.9 eV and (c) 2.15 eV, represented with solid and dotted curves, respectively.

## Chiral Emission Measurements

To characterize the degree of circular polarization, we measured the circularly polarized PLE maps by placing a quarter-wave plate and a linear polarizer in front of the detector (Figure  S2). Using the PLE maps of right-handed circular polarization (RCP) and left-handed circular polarization (LCP), we determined the *g*_PL_ maps, as illustrated in Figure 3. For the nondisplaced sample, the *g*_PL_ value is zero due to its achiral structure (Figure  S4). In addition, the mirror symmetry between positively and negatively displaced rods in the unit cell results in *g*_PL_ dispersion maps with opposite signs and similar magnitudes (note that the left and right panels of Figure 3a correspond to the dispersion of *g*_PL_ for negatively and positively displaced rods, respectively). For the emission along the *x*-axis, the SLR mode produces a significant |*g*_PL_| of approximately 0.15, while the quasi-BIC mode exhibits a relatively smaller |*g*_PL_| value of approximately 0.09 at its peak. It is worth noting that the *g*_PL_ of the quasi-BICs varies slightly with incident angle due to the contribution of extrinsic chirality.^55^ For the emission along the *y*-axis (Figure  S5), the SLR peak exhibits a similar |*g*_PL_| ≈ 0.2, while the low-dispersion mode shows a maximum |*g*_PL_| ≈ 0.1. Interestingly, in both emission directions, the *g*_PL_ measured at 1.9 eV has the opposite sign of the *g*_PL_ measured at 2.15 eV (Figure 3b,c). Note that the spectral overlap between photonic modes and molecular absorption spectra does not influence *g*_PL_ since molecular absorption is too weak to alter the near-field electric field distribution under the weak coupling regime. In addition, a similar array structure made of metallic materials has exhibited higher *g*_PL_ under high excitation powers due to nonlinear (lasing) emission,^37^ in contrast to the present study, which is conducted in the linear response regime.

**Figure 3:**
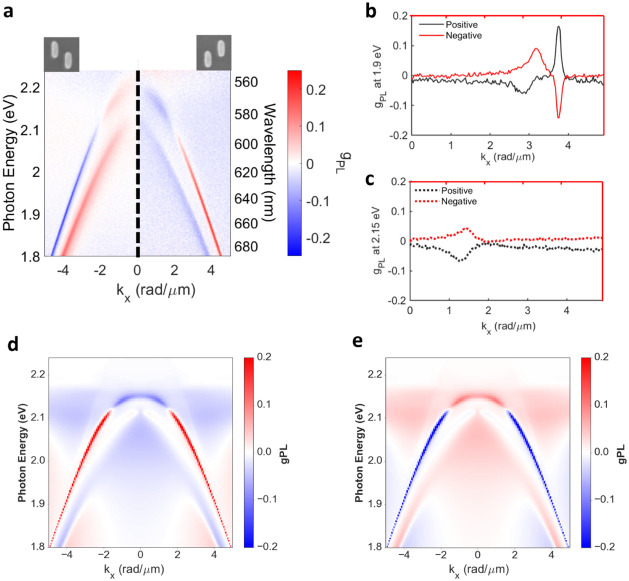
PL dissymmetry factor of the chiral metasurfaces with a 220 nm thick layer of the dye-doped polymer on top. *g*_PL_ maps as a function of photon energy (wavelength) and *k*_*x*_ for the array of positively displaced Si nanorods in the *y*-direction (right panel) and the negatively displaced Si nanorods (left panel). *g*_PL_ as a function of *k*_*x*_ at (b) 1.9 eV and (c) 2.15 eV. Calculated PL dissymmetry maps of (d) the positively displaced rods and (e) the negatively displaced rods radiating along the *x*-axis with a 220 nm dye-doped polymer film.

## Discussion

To elaborate on the far-field emission enhancement, we performed numerical calculations based on COMSOL. The PL dissymmetry factor is obtained from the scattering problem illuminating with circularly polarized light and invoking Kirchhoff’s law, as explained in the Methods section. The resulting *g*_PL_ bands for different frequencies and angles of incidence are shown in Figure 3d,e. The agreement with the experimental measurements is notable, showing only a slight frequency shift. Note that imperfections in the particle shape, such as the rounding of the edges, can cause subtle energy shifts while having little effect on *g*_PL_ (Figure  S6).

In order to shed light on the chirality of the modes, a multipolar decomposition of the induced near-fields by the metasurfaces is also carried out through COMSOL (see Methods section). We only consider the contribution of the electric (**p**) and magnetic (**m**) dipole moments, since previous studies indicate that the quadrupolar response extends beyond the spectral region we focus on.^55^ For simplicity, we only analyzed the results of the positively displaced rods, since the physical interpretation in the negatively displaced rods must be symmetric. We analyzed the electric and magnetic dipoles of each rod: (**p_1_**, **m_1_**) and (**p_2_**, **m_2_**). However, according to the *C*_2_ (inversion) symmetry of the system, it is sufficient to analyze the magnitude of the dipoles in one rod, since *p* = |**p_1_** | = |**p_2_** | and *m* = |**m**_1_| = |**m**_2_|, and the symmetric/antisymmetric character of the pair, as the only two possibilities are **p**_1_ = **p**_2_ and **p**_1_ = **–p**_2_ for the electric dipoles, and **m**_1_ = **m**_2_ and **m**_1_ = **–m**_2_ for the magnetic dipoles. Both *p* and *m* are presented in units of Coulomb times meter (C·m) for direct comparison. For this, *m* is normalized by the factor *n/c*, where *n* is the refractive index of the medium surrounding the metasurface and *c* is the speed of light in vacuum. The results for the multipolar contributions to reflection upon illumination with a wavevector along the *x*-axis are presented in Figure 4a for the case of the positively displaced rods. We exploit the dipolar symmetries mentioned above, plotting the magnitude of the dipolar contributions along the Cartesian directions for a single nanorod, encoding in the color the symmetric/antisymmetric character in the dimer: the magenta color scale indicates the contribution of symmetric dipoles per unit cell, while the blue/green color scale indicates the contribution of antisymmetric dipoles. Figure 4a shows that the quasi-BIC mode is associated with a significant in-plane antisymmetric *p*_*y*_ mode and a weak out-of-plane symmetric *m*_*z*_, both nonradiative at *k* = 0. The resulting π-rotation symmetry confirms the BIC character at the Γ-point with a diverging *Q*-factor, which supports its high PLE along the quasi-BIC band. The direction of **p** is verified by the experimental extinction maps, showing that the quasi-BIC mode is associated with the excitation polarization along the *y*-axis (Figure  S7). The antisymmetric *p*_*y*_ character of the dimer in turn supports the emergence of positive (respectively, negative) *g*_PL_ for negatively (respectively, positively) displaced nanorods in Figure 3a. On the other hand, the SLR mode arises from the out-of-plane antisymmetric *p*_*z*_ together with a strong in-plane symmetric *m_x_* contribution. This mode cannot become a BIC at the Γ-point due to the lack of π-rotation symmetry within the unit cell and the in-plane character of the *m*_*x*_ contribution. Note that the significant *m*_*x*_ in the SLR mode explains its high *g*_PL_, with different signs depending on the displacement of the dimer rods (Figure 3a).

**Figure 4:**
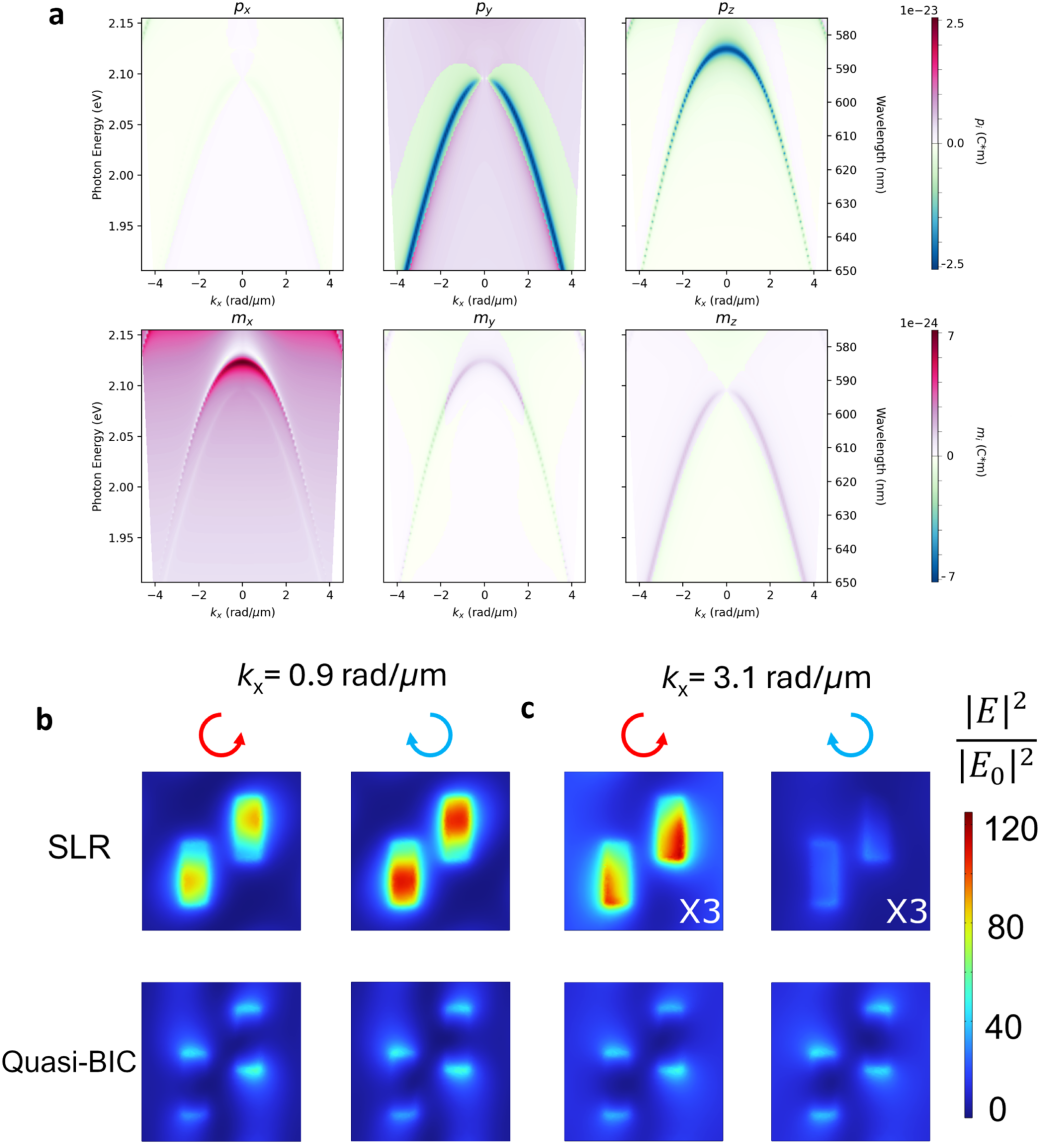
(a) Angle-dispersive mode contribution maps simulated along the *k*_*x*_ axis of the array of positively displaced Si nanorods with the dye film thickness of 220 nm. Upper panels: electric dipole components along the *x, y*, and *z* directions; lower panels: magnetic dipole components along the *x, y*, and *z* directions. The magenta color scale indicates the contribution of symmetric dipoles per unit cell, while the blue/green color scale indicates the contribution of antisymmetric dipoles. (b,c) Electric near-field distribution of the SLR mode and the quasi-BIC mode on the *xy* plane at a height of 10 nm over the top of the rods with a 220 nm dye-doped polymer film with an illumination at (b) *k*_*x*_ = 0.9 rad/μm (2.11 eV) and (c) 3.1 rad/μm (1.98 eV).

To investigate the mechanism leading to the sign flip of *g*_PL_ for the SLR, we analyze the near-field distribution of the electric field when illuminated with RCP and LCP light. Note that the Lorentz reciprocity theorem relates the outcoupling efficiency to the near-field intensity.^56,57^ Figure 4b,c shows the near-field distribution of the SLR on the *xy* plane at a height of 10 nm above the top of the rods. For |*k_x_* | = 0.9 rad/μm and a photon energy of 2.11 eV (Figure 4b), the field intensity with RCP illumination is stronger than that with LCP illumination. The field intensity with RCP illumination becomes weaker than that with LCP illumination when |*k_x_* | = 3.1 rad/μm and a photon energy of 1.98 eV (Figure 4c). These results verify that the same SLR mode could possess different handedness under different incident photon energies (and corresponding in-plane wavevectors). In comparison, the field distributions of the quasi-BIC mode are similar for different *k*_*x*_ and photon energies, indicating that this mode preserves its multipolar character and symmetries along the entire band.

To further support the multipolar-based arguments, we evaluate the helicity density (*h*) maps based on the eigenmodes corresponding to the quasi-BIC and SLR (see Methods section and Figure  S8), showing both in-plane and out-of-plane (plane of incidence) cuts: the *xy* plane crossing the center of the Si rods, and the *xz* plane crossing the center of the unit cell. A uniform helicity, mostly concentrated inside, is observed along the rods for the quasi-BIC, with antisymmetric features being very weak outside the rods. On the other hand, the SLR exhibits a more complex helicity pattern even inside the rods. A clear antisymmetric pattern along the *z* direction is observed inside each rod, compatible with a strong circulation of the electric field in an *yz* plane originating an effective *m_x_* contribution. Note also that larger helicity densities are found between the rods.

We also explored the impact of the dye layer thicknesses in the *g*_PL_. For this investigation, we prepared a sample with a 380 nm thick dye-doped layer on the same arrays. The corresponding PLE dispersion maps for *k*_*x*_ and *k*_*y*_ are shown in Figure S9, showing a weaker PLE (≈3–5 ) and a slightly larger *Q* factor ≈94 in the quasi-BIC mode than the one measured with a thinner dye layer (PLE ≈ 5–8) (see Figure  S3). Incidentally, a waveguide mode arises in the dye layer for this dye layer thickness near the quasi-BIC and SLR band frequencies; however, we have verified that it lies at larger *k*_*x*_ wavevectors upon folding their dispersion relations into the first Brillouin zone to account for array diffraction. The resulting *g*_PL_ maps are shown in Figure 5a for both positively and negatively displaced nanorods. Apart from a nonzero background signal, similar quasi-BIC and SLR bands are observed, yielding significant *g*_PL_ values, along with the new guided mode at higher frequencies beyond the folded diffraction line. The corresponding numerical simulations are shown in Figure  S10, which agree well with the behavior of the experimental PL dissymmetry factors. Furthermore, the analysis of the mode decomposition (Figure  S11) reveals that the dipole characters of the quasi-BIC mode and the SLR mode are preserved for the thicker layer. Incidentally, the waveguide mode is associated with a significant symmetric *m*_*x*_ component. The helicity densities also agree with the multipolar analysis on the nature of both bands (Figure  S8). The main difference is in the SLR helicity, which is weaker for the thicker layer, revealing, in turn, a weaker perpendicular confinement and uniform sign. In comparison, the quasi-BIC mode remains essentially unchanged upon variation of the layer thickness. The most relevant feature is that no sign flip of *g*_PL_ in the SLR mode is observed in Figure 5a for the case of the layer 380 nm thick, unlike that seen for the 220 nm thick case (at |*k_x_* | > 2 rad/μm in Figure 3a). According to the near-field distributions (Figure 5,e), similar relative intensities are obtained for LCP/RCP illumination at different energies throughout the SLR band, confirming that the sign of *g*_PL_ remains constant. A possible explanation for the robust values of *g*_PL_ with layer thickness for the quasi-BIC mode compared to the SLR could be the stronger lateral field confinement,^54^ also evidenced in our helicity density calculations shown in Figure  S8. The quasi-BIC mode is sensitive only to changes very close to the nanoparticles, so the change in layer thickness does not modify its nature. On the other hand, the SLR mode, which is closer to the diffraction line and less confined, is more sensitive to the distance to the interface, thus showing changes in the *g*_PL_ sign for thin enough layers. Another possible explanation for the robustness of the quasi-BIC mode compared to the SLR lies in their local/nonlocal characteristics.^58^ This is supported by the larger full width at half-maximum of the *g*_PL_ of the quasi-BIC compared with that of the SLR, which indicates greater dissipation due to scattering and outcoupling through localized resonances (Figures 3a and 5a). Owing to the more localized nature of the quasi-BIC mode, its chirality is less sensitive to extrinsic effects and to the layer thickness.

**Figure 5:**
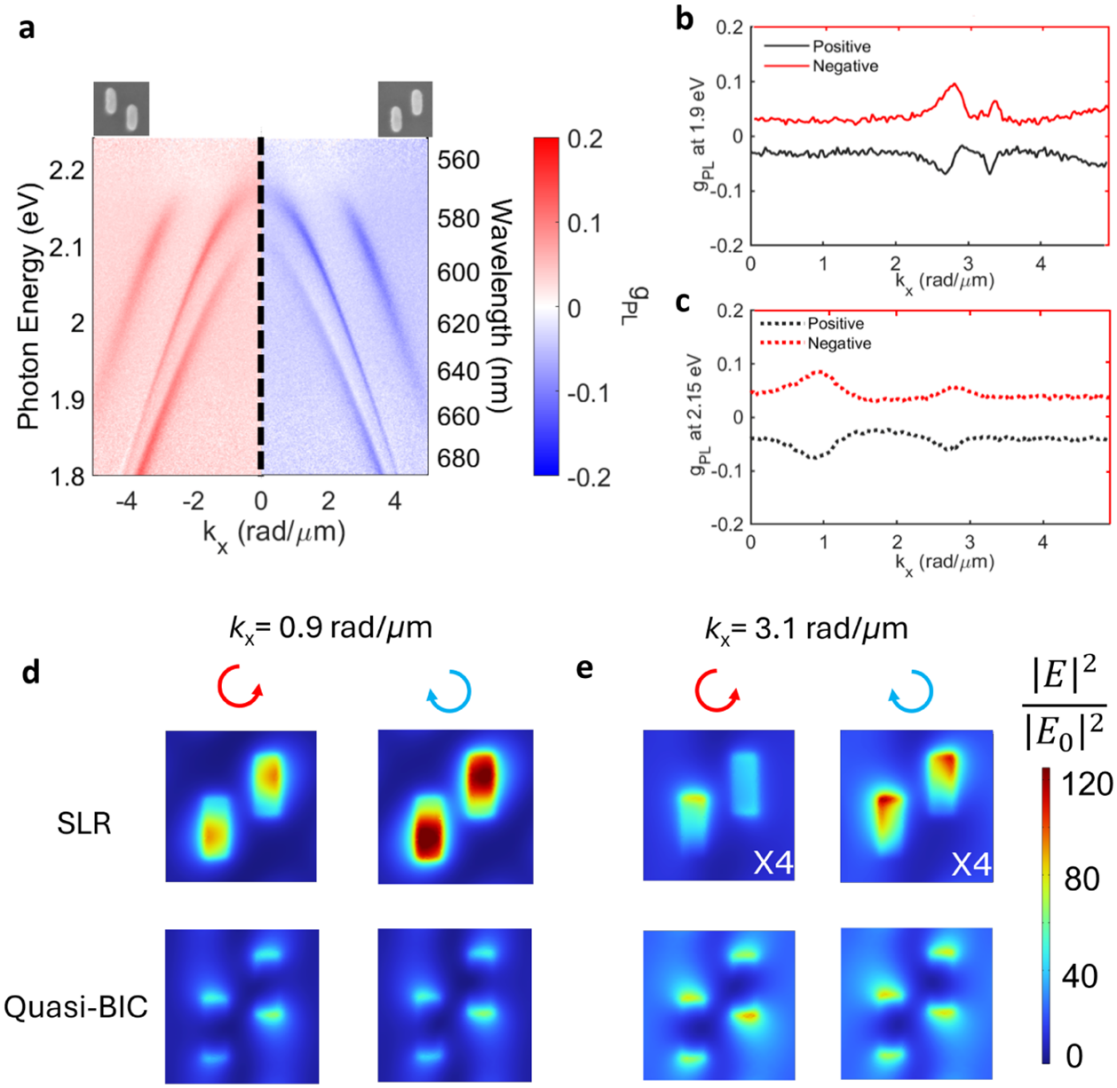
PL dissymmetry factors of the chiral metasurfaces spin-coated with a 380 nm thick dye-doped polymer film. (a) *g*_PL_ dispersion maps as a function of the photon energy and *k*_*x*_ for the metasurface with positively displaced rods (right) and negatively displaced rods (left). (b) *g*_PL_ as a function of *k*_*x*_ at 1.9 eV, and (c) 2.15 eV. Electric near-field distribution of the SLR mode and the quasi-BIC mode on the *xy* plane at a height of 10 nm over the top of the rods with a 380 nm dye-doped polymer film with an illumination at (d) *k*_*x*_ = 0.9 rad/μm (2.11 eV) and (e) 3.1 rad/μm (1.98 eV).

## Conclusions

The mechanism of CPL generation from quasi-BICs and SLRs in intrinsic chiral Si metasurfaces has been investigated. We have characterized the photoluminescence enhancement and *g*_PL_ in metasurfaces with organic dye layers of different thicknesses (Table 1). The quasi-BIC mode exhibits higher PLE ≈ 4.9 and *Q* factor ≈ 69 for a layer thickness of 220 nm, but relatively low values of *g*_PL_ ≈ 0.09, which are robust under the variation of *k*_*x*_ and dye thickness. For the SLR mode under a layer thickness of 220 nm, higher *g*_PL_ ≈ 0.15 with lower PLE ≈ 1.8 and *Q* factor ≈ 32 are achieved. In addition, the sign of *g*_PL_ is inverted under different *k*_*x*_ and dye thicknesses. Our theoretical analysis based on numerical calculations reveals that the quasi-BIC mode is dominated by strong in-plane antisymmetric electric dipole (**p**) contributions, leading to significantly enhanced PLE, whereas the SLR mode features pronounced magnetic dipole (**m**) components that contribute to a higher *g*_PL_, with much lower PLE though. Furthermore, the quasi-BIC mode maintains an invariant spatial distribution of the electric field and helicity density under environmental changes, underscoring its exceptional robustness. In contrast, the SLR mode exhibits significant variation in field distributions under similar changes, leading to the handedness flipping of CPL. To further increase the PLE and *g*_PL_, low-loss dielectric materials such as Si_3_N_4_ or TiO_2_ can be employed as the constituent material for the nanoparticles in metasurfaces.^59^ In addition, since the spectral overlap between the emission spectra of dyes and photonic modes only affects how the photonic modes are projected in PL, the robustness and inversion of *g*_PL_ are independent of the dye characteristics, provided that their emission overlaps with the photonic modes. These findings elucidate the distinct chiro-optical characteristics of quasi-BIC and SLR modes in intrinsic chiral silicon metasurfaces, with the quasi-BIC mode standing out for its chiro-optical robustness and offering strategic insights for optimizing CPL performance in metasurface-based luminescent devices.

**Table 1:** PLE, *Q* Factor, and *g*_PL_ of SLRs and Quasi-BICs^a^

	Thickness	SLRs	quasi-BICs
PLE	220 nm	1.8 ± 0.1	4.9 ± 0.8
	380 nm	1.2 ± 0.05	2.8 ± 0.2
*Q* factor	220 nm	32 ± 7	69 ± 2
	380 nm	113 ± 19	94 ± 6
$\left|g_{\mathrm{PL}}\right|$	220 nm	0.15 ± 0.02	0.09 ± 0.03
	380 nm	0.08 ± 0.01	0.09 ± 0.04

a PLE and *g*_PL_ are obtained from the measurements in Figures 2a, S9, and 3b at an energy of 1.9 eV. The *Q* factor is obtained from Figure 2a at *k*_*x*_ = 3.1 rad/μm. The deviations arise from the measurements of the arrays with positively and negatively displaced nanorods .

## Methods

### Fabrication of Si Metasurfaces

Polycrystalline Si thin films, 90 nm in thickness, were deposited on a synthetic silica glass substrate by using low-pressure chemical vapor deposition with SiH_4_ gas as the Si source. A positive resist (ZEP520A) was applied to the Si film and subjected to electron-beam lithography. Subsequently, nanorod hole arrays of resist were formed on the Si film through development. Next, a Cr layer (70 nm, by electron-beam deposition) was deposited on the hole array, and a liftoff process resulted in the Cr nanorod arrays on the Si film. Using Cr as a mask, the Si film was then vertically etched by a selective dry etching process (Bosch process) using SF_6_ and C_4_F_8_ gases. Finally, the Cr mask was removed through wet etching in an acidic solution (S-clean S24, Sasaki Chemical Co., Ltd.). The resulting array covered an area of 2 × 2 mm^2^.

### Fourier Microscope

Figure  S2 shows a schematic illustration of the Fourier microscope used to image the angular distribution of PL, which is measured as a function of the photon energy and in-plane wavevectors *k*_x_ and *k*_y_ is, which are related to the outcoupling polar angle of the emission from the sample. An optical diffuser is placed in front of the sample to create unpolarized excitation from the incident beam. An illumination objective is used to excite the sample within the area of the particle array. When the PL from the sample passes through a collection objective, each wavevector component is separated and focused at a different position in the back focal plane (BFP), creating a Fourier image. To measure the CPL, a quarter-wave plate (QWP) and a linear polarizer are placed after the collection objective. Two lenses are used to image the BFP onto the detector. These two lenses are positioned at a focal length after the QWP, increasing the working distance.

### COMSOL Simulations

The multipole decomposition and calculation of the near-fields of the modes were performed with the Electromagnetic Waves in Frequency Domain module of COMSOL Multiphysics. The simulation was done for the Si array covered with perylene dye molecules in PMMA. We calculated the polarization **P** induced in the system by external plane waves impinging at different angles with TE polarization. The multipole decomposition of the modes can be calculated by integrating **P**:^60–63^

1 $$p=\int Pj_{0}\left(kr\right)d^{3}r+\frac{k^{2}}{2}\int \left\{3\left(r\cdot P\right)r-r^{2}P\right)\}\frac{j_{2}\left(kr\right)}{{\left(kr\right)}^{2}}d^{3}r$$

2 $$m=-\frac{i3\omega }{2}\int \left(r\times P\right)\frac{j_{1}\left(kr\right)}{kr}dr$$

where ω is the optical frequency. Higher-order contributions are negligible in the spectral range.

The helicity density is defined as

3 h(r)=−12ε0μ0ωIm{E*·B}

where ε_0_ and μ_0_ are the vacuum permittivity and vacuum permeability, respectively. The vectors **E** and **B** denote the electric and magnetic fields, respectively.

### Simulations of Angle-Dependent *g*_PL_ Maps

Based on the Lorentz reciprocity theorem for the light out-coupling efficiencies associated with the near-field enhancements,^57,64^ we calculated the near-field electric field intensity in the organic layer. To obtain *g*_PL_, we used LCP and RCP as the light sources to illuminate the metasurface. The molecular dipole orientations are assumed to be fully random. We obtained *g*_PL_ as a function of *k* and λ by evaluating the field intensity difference between LCP and RCP excitations as follows.^65,66^

4 $$g_{\mathrm{PL}}\left(k,\lambda \right)=2{∭}_{V}\frac{\left|E_{\mathrm{LCP}}\left(r,k,\lambda \right){|}^{2}-\right|E_{\mathrm{RCP}}\left(r,k,\lambda \right){|}^{2}}{\left|E_{\mathrm{LCP}}\left(r,k,\lambda \right){|}^{2}+\right|E_{\mathrm{RCP}}\left(r,k,\lambda \right){|}^{2}}dr^{3}$$

where *k* and λ are the in-plane wave vector and emission wavelength, respectively. **E**_LCP_(**r**, *k*, λ) and **E**_RCP_(**r**, *k*, λ) are the near-electric field at position **r** for wave vector *k* and wavelength λ with LCP and RCP excitation, respectively. The integrated volume *V* covers the entire organic layer.

## Supplementary Material



## Notes

Absorption and emission spectra of the dye (Figure S1); setup of Fourier microscope (Figure S2); PLE along the *y-*axis for all samples with layer thickness of 220 nm (Figure S3); *g*_PL_ of the nondisplaced sample (Figure S4); *g*_PL_ along the *y-*axis for all samples with layer thickness of 220 nm (Figure S5); shape variation effects on PLE and *g*_PL_ (Figure S6); extinction dispersion maps for all samples with layer thickness of 220 nm (Figure S7); helicity density map analysis (Figure S8); PLE along the *x* and *y* axes for all samples with layer thickness of 380 nm (Figure S9); simulated *g*_PL_ maps with layer thickness of 380 nm (Figure S10); simulated angle-dispersive mode deposition maps with layer thickness of 380 nm (Figure S11) (PDF)
