# Supplementary material for: Robust Circularly Polarized Luminescence via Quasi-Bound States in the Continuum in Intrinsic Chiral Silicon Metasurfaces
Source: ACS Photonics. 2025 Oct 27;12(11):6428–37. doi: 10.1021/acsphotonics.5c01966 (PMC12637853; doi:10.1021/acsphotonics.5c01966)
Supplement: Supplementary file 1 [file ph5c01966_si_001.pdf]

Supporting Information: Robust Circularly Polarized Luminescence via Quasi-Bound States in the Continuum in Intrinsic Chiral Silicon Metasurfaces

Xiao-ke Zhu,[†,‡] Yu-Chen Wei,[*,†] Jose L. Pura,[¶,§] Matthijs Berghuis,[†] Minpeng Liang,[†] Beatriz Castillo López de Larrinzar,[‖,¶] Shunsuke Murai,[⊥] Antonio García-Martín,[‖] José A. Sánchez-Gil,[¶] Sailing He,[‡] and Jaime Gómez Rivas[*,†]

†*Department of Applied Physics and Science Education, Eindhoven University of Technology, 5600MB Eindhoven, The Netherlands*

‡*Centre for Optical and Electromagnetic Research, National Engineering Research Center for Optical Instruments, Zhejiang University, Hangzhou 310058, China*

¶*Instituto de Estructura de la Materia (IEM-CSIC), Consejo Superior de Investigaciones Científicas, Serrano 121, 28006 Madrid, Spain.*

§*GdS-Optronlab, Física de la Materia Condensada, Universidad de Valladolid, Paseo de Belén 19, 47011 Valladolid, Spain.*

‖*Instituto de Micro y Nanotecnología IMN-CNM, CSIC, CEI UAM+CSIC, Isaac Newton 8, E-28760 Tres Cantos, Madrid, Spain.*

⊥*Department of Electronics and Physics, Graduate School of Engineering, Osaka Metropolitan University, Osaka, 599-8531, Japan*

E-mail: y.c.wei@tue.nl; j.gomez.rivas@tue.nl

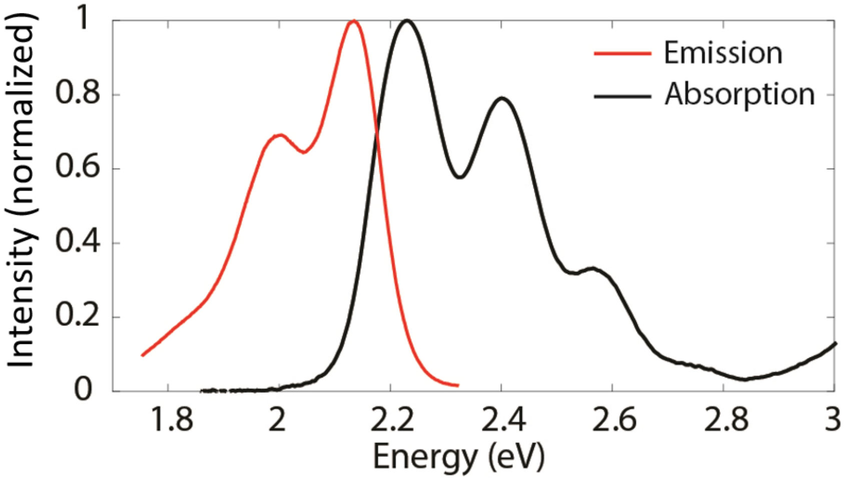

Figure S1: Normalized absorption and emission spectra of the perylene dye in PMMA.

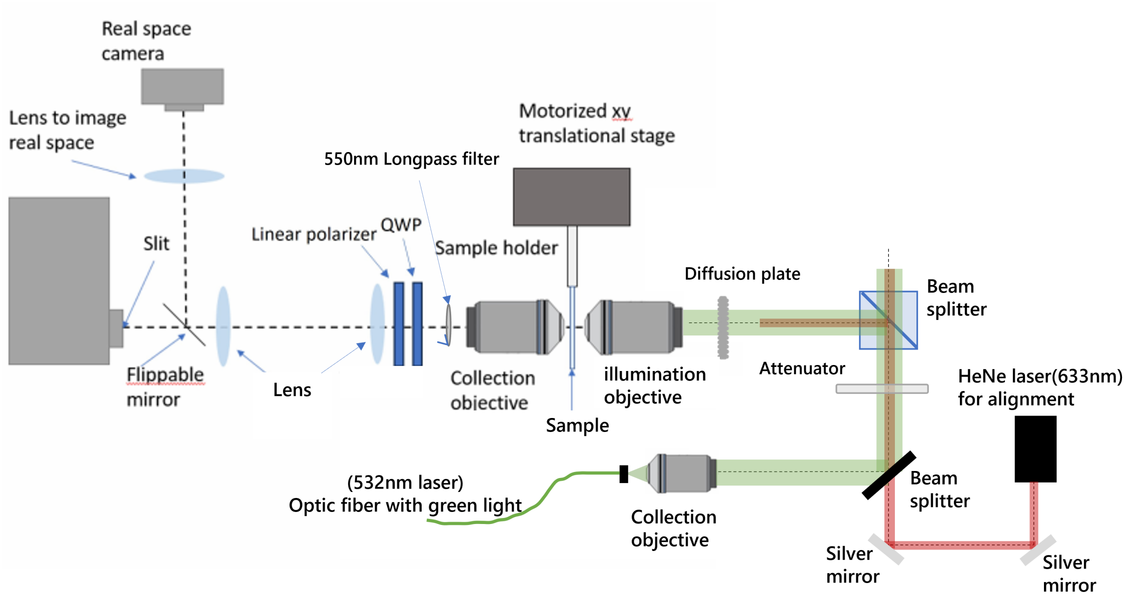

Figure S2: Fourier microscope for dispersion measurement of the PL with a quarter-wave plate (QWP) and a linear polarizer to detect circular polarized light. A more detailed description of the setup can be found in the reference.[1]

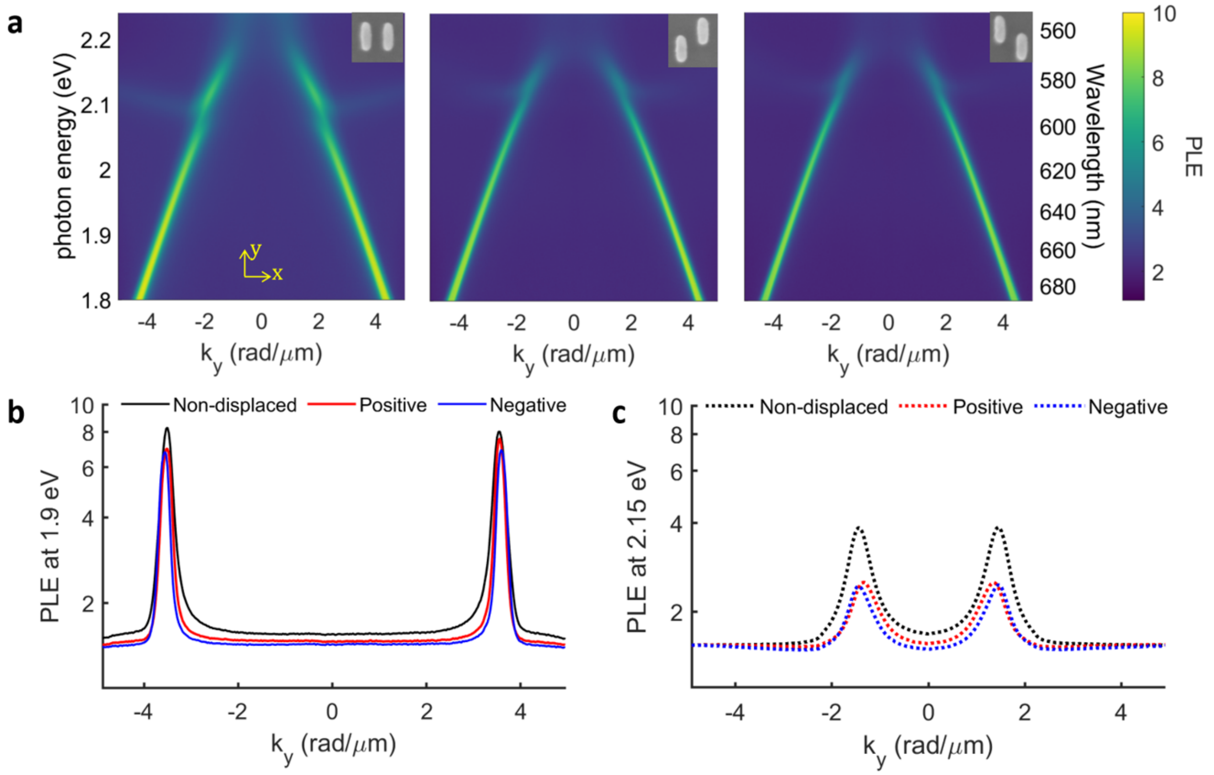

Figure S3: PL analysis for three metasurfaces of nanorod dimers with a spin-coated dye-doped polymer film with a thickness of 220 nm on top. (a) PLE for metasurfaces with an unit cell formed by (left) two equal non-vertically displaced Si nanorods, (middle) two positively displaced Si nanorods in the y-direction, and (right) two negatively displaced Si nanorods in the y-direction, measured with a Fourier microscope as the function of the in-plane wave vector k_y. The insets show the SEM images of the unit cell for each structure. PLE as the function of k_y at (b) 1.9 eV and (c) 2.15 eV represented with solid and dotted curves, respectively.

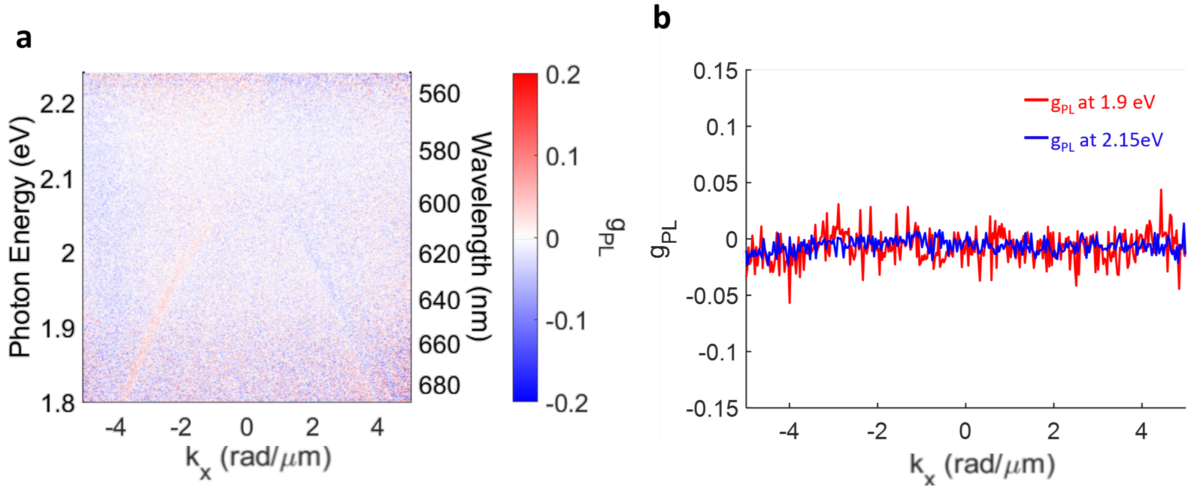

Figure S4: PL dissymmetry (presented as g_{PL}) of the non-displaced nanorods metasurface with a 220 nm dye-doped polymer film. (a) g_{PL} maps as function of photon energy and k_x. (b) g_{PL} as the function of k_x monitored at 1.9 eV and 2.15 eV along the x axis.

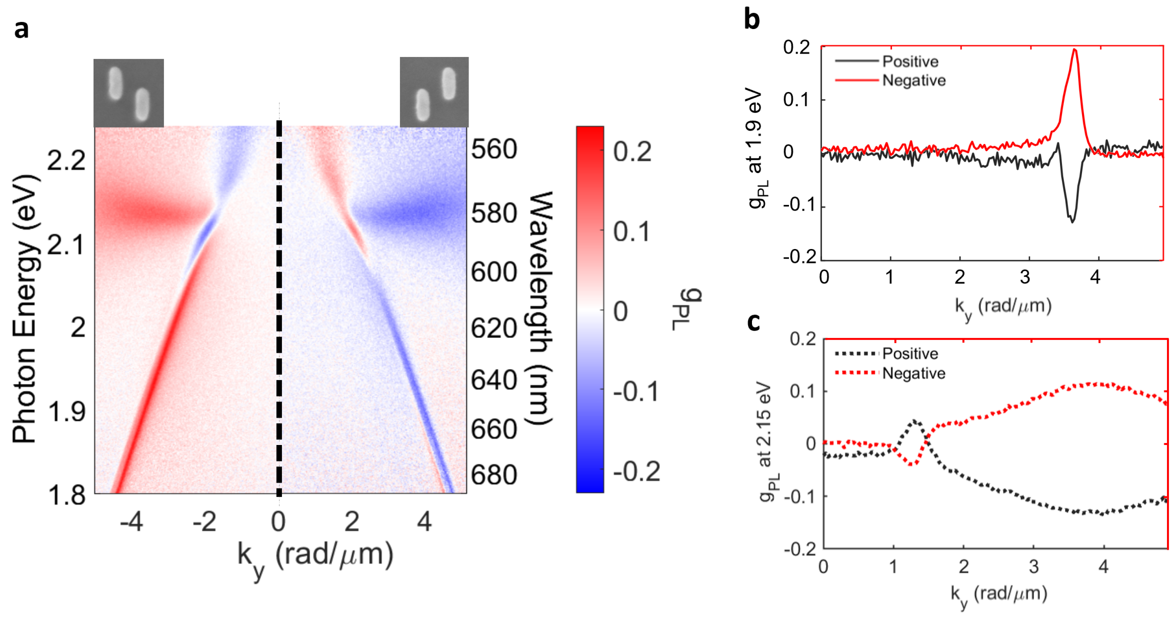

Figure S5: PL dissymmetry (presented as g_{PL}) of chiral metasurfaces spin-coated with a 220 nm dye-doped polymer film. g_{PL} maps as a function of photon energy (wavelength) and k_y for the array of positively displaced Si nanorods in the y-direction (left), and the negatively displaced Si nanorods (right). g_{PL} as the function of k_y monitored at (b) 1.9 eV and (c) 2.15 eV along the y axis.

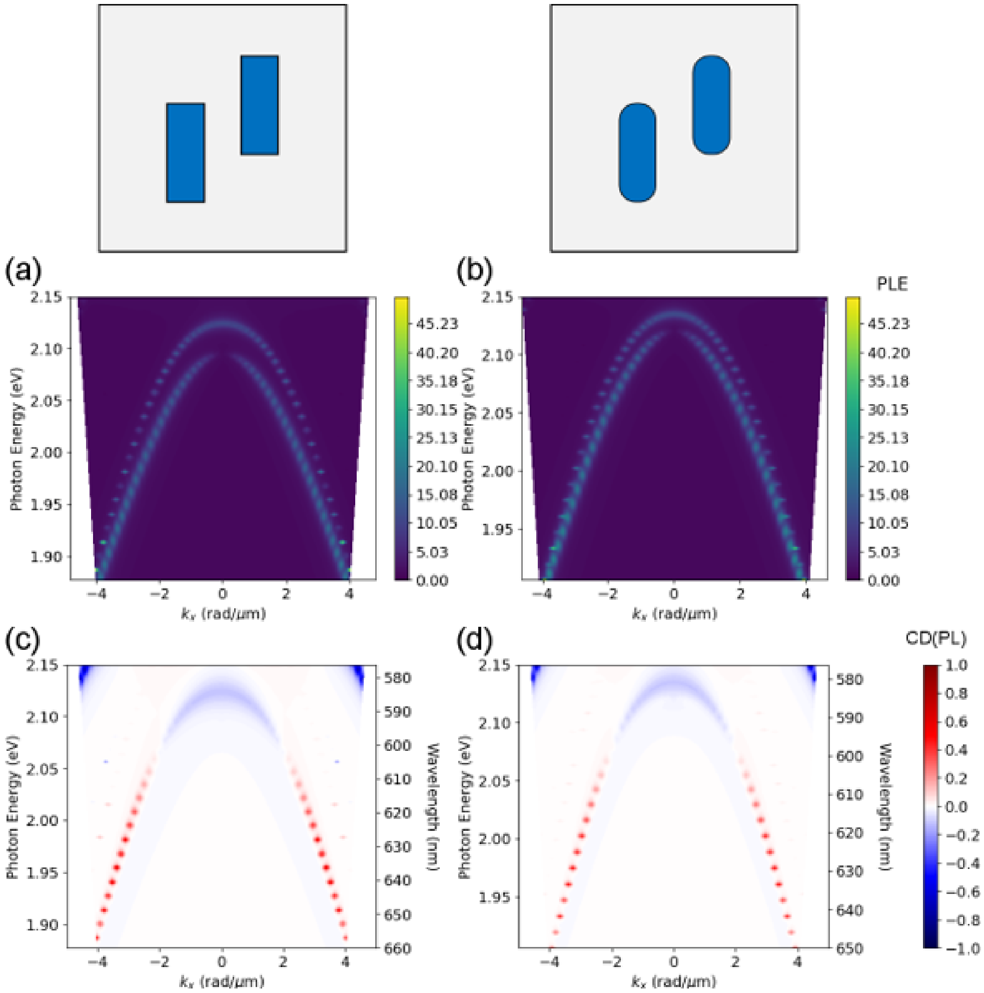

Figure S6: Shape variation effects on PLE and dissymmetry factors. (a-b) PLE calculated with COMSOL for rectangular and elliptical nanorods, respectively. The curvature radius is selected to be half the rod width: $R = L_x/2$. (c-d) Circular dichroism of the expected photoluminescence (CD(PL)) calculated with COMSOL for rectangular and elliptical nanorods, respectively.

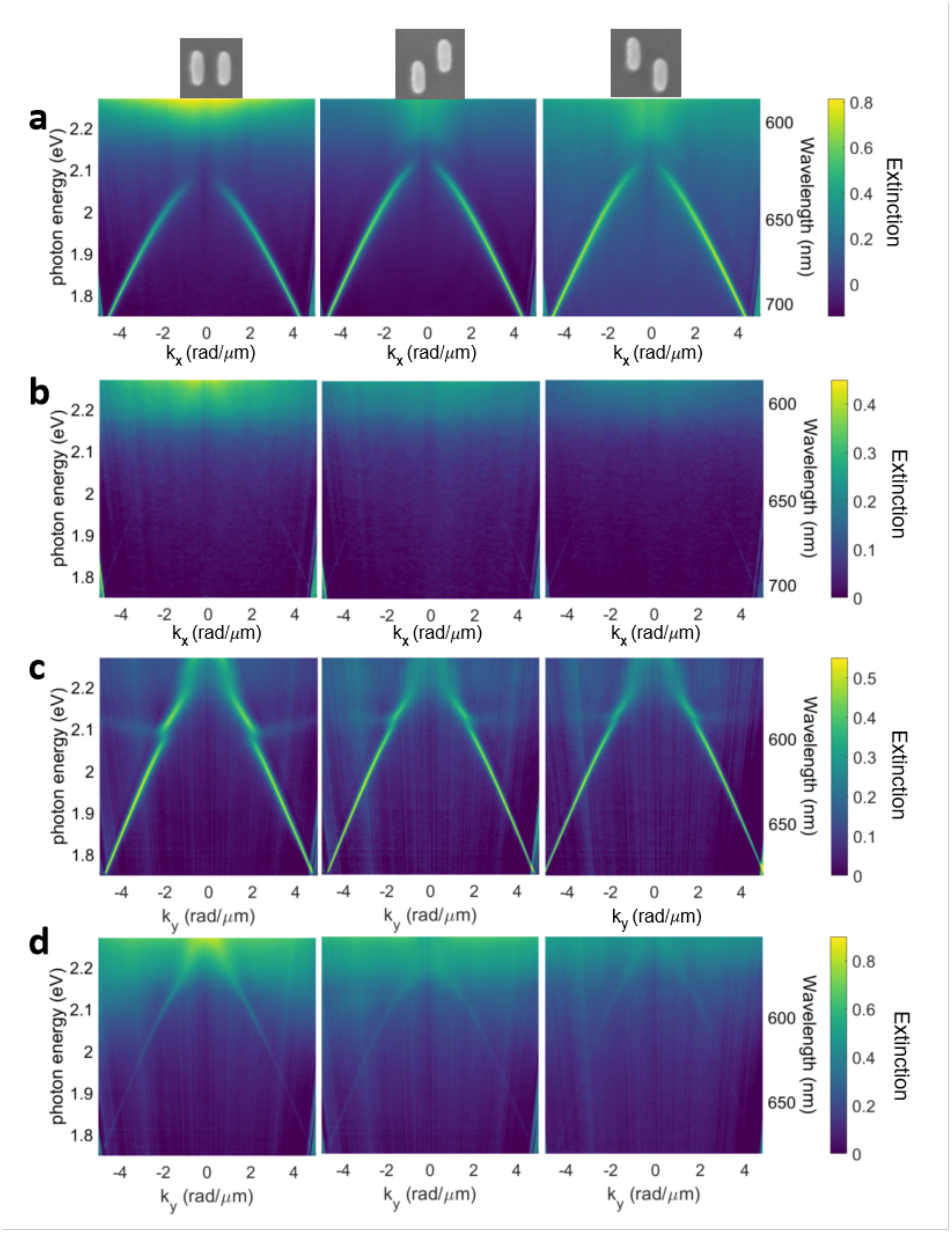

Figure S7: Extinction maps for three types of metasurfaces with the dye-doped polymer film on top (220 nm) (a) illuminated with the x-polarized light as a function of k_x, (b) illuminated with the y-polarized light as a function of k_x, (a) illuminated with the y-polarized light as a function of k_y and (d) illuminated with the x-polarized light as a function of k_y.

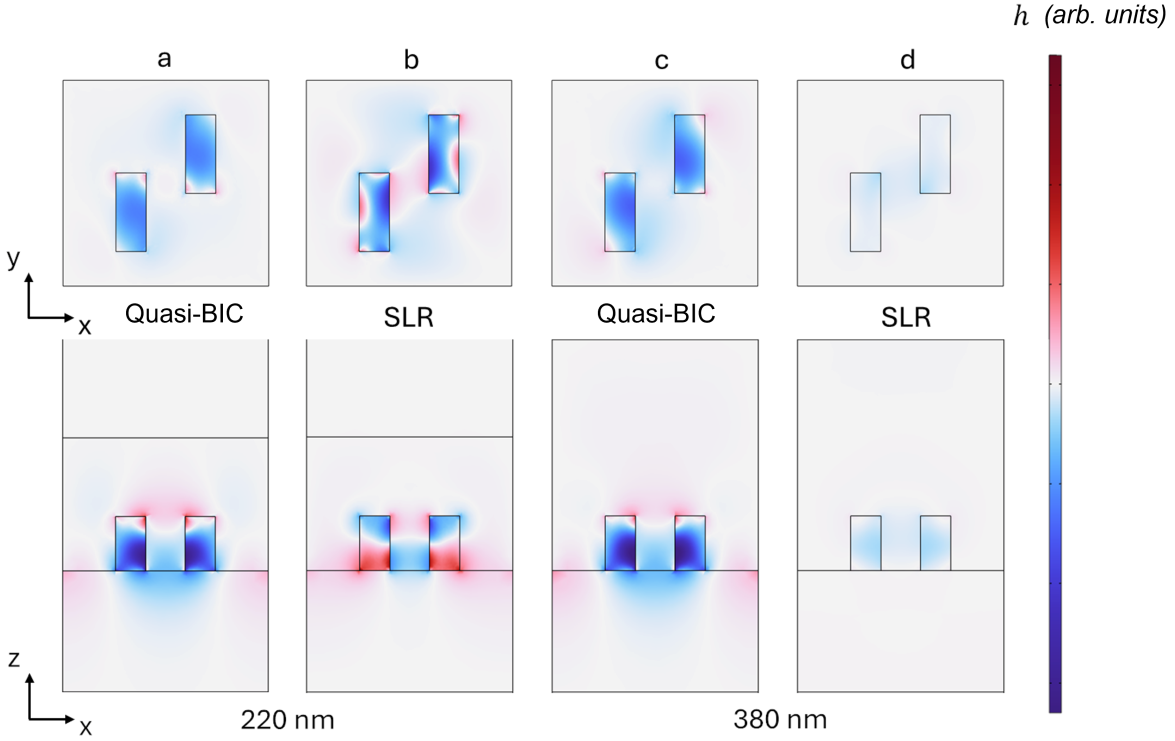

Figure S8: Helicity density maps of (a) the SLR mode and (b) the quasi-BIC mode with layer thickness 220 nm. Helicity density maps of (c) the SLR mode and (d) the quasi-BIC mode with layer thickness 380 nm.

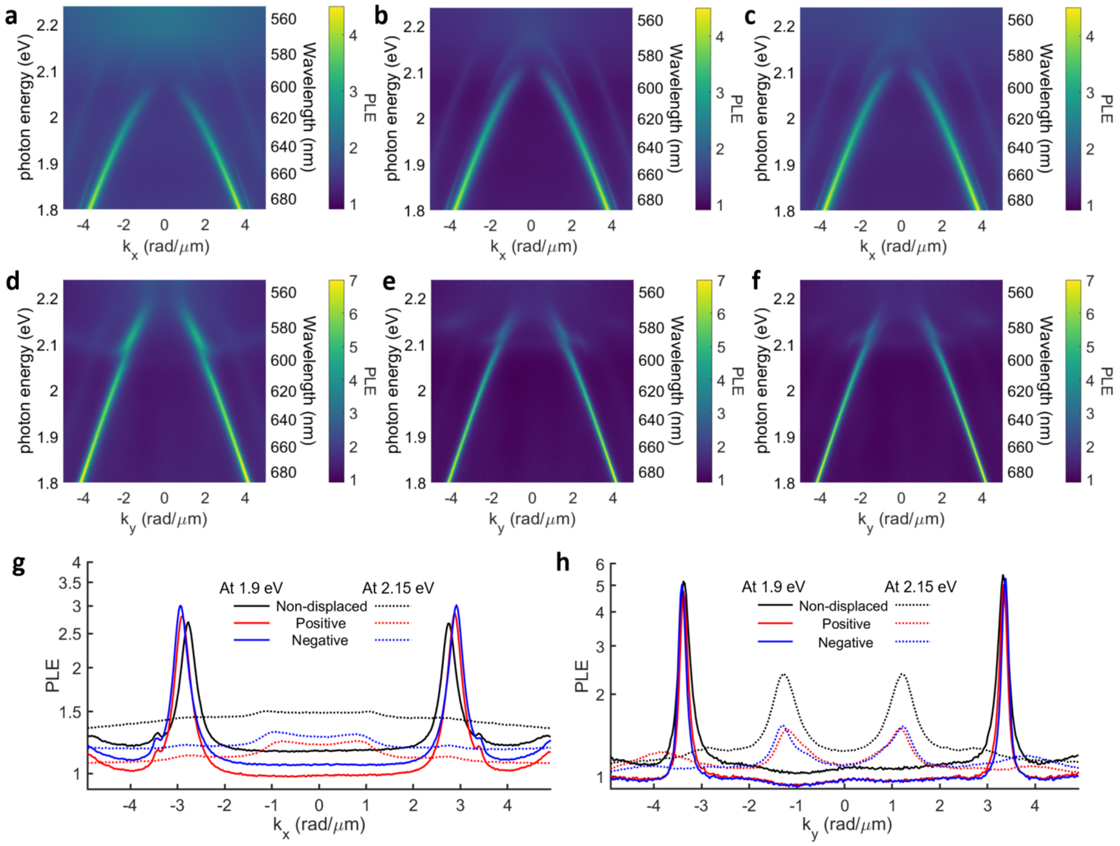

Figure S9: Photoluminescence (PL) analysis for three metasurfaces of different nanorod dimers spin-coated with a 380 nm dye-doped polymer film. (a-c) Photoluminescence enhancement (PLE) for metasurfaces with (a) non-displaced nanorod dimer, (b) positively displaced nanorod dimer, and (c) negatively displaced nanorod dimer as the function of k_x.(d-f) The PLE for metasurfaces with (a) non-displaced nanorod dimer, (b) positively displaced nanorod dimer, and (c) negatively displaced nanorod dimer as the function of k_y. (g) PLE as the function of k_x at 1.9 eV and 2.15 eV represented with solid and dotted curves, respectively. (h) PLE as the function of k_y at 1.9 eV and 2.15 eV represented with solid and dotted curves, respectively.

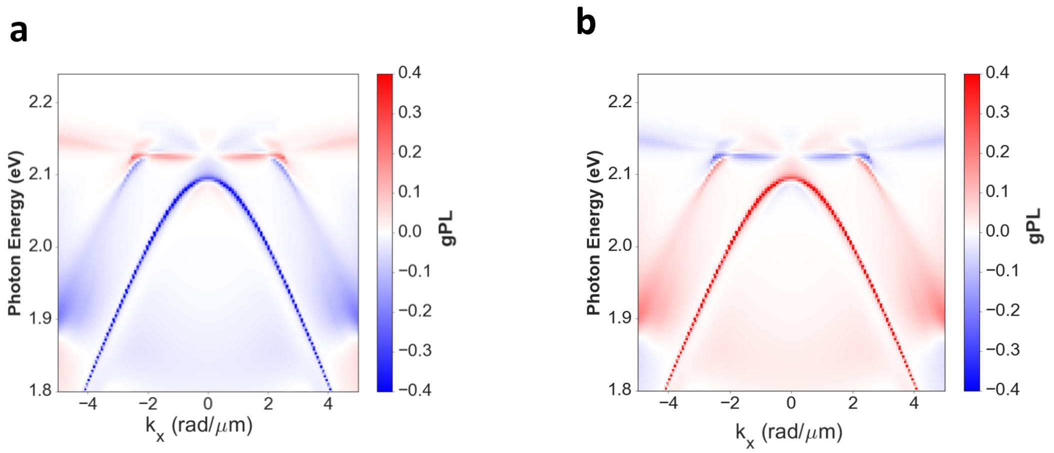

Figure S10: Simulated PL dissymmetry maps of (a) the positively displaced nanorod metasurface, and (b) the negatively displaced nanorod metasurface as a function of k_x with a 380 nm dye-doped polymer film on top.

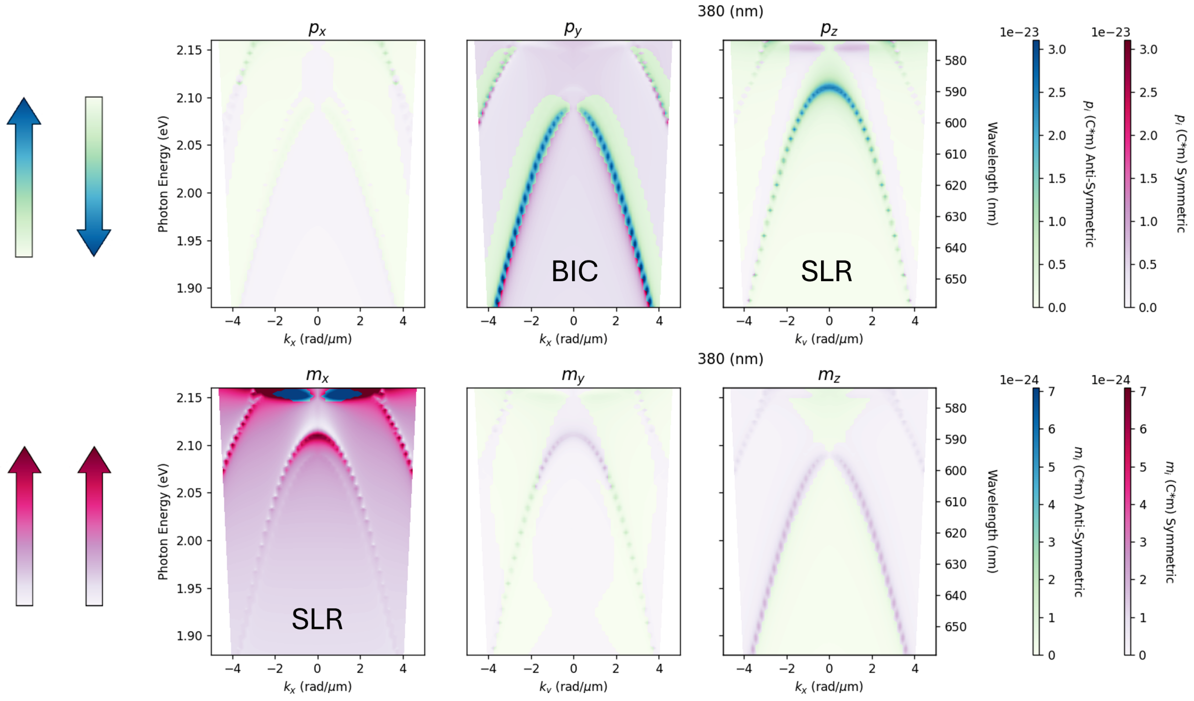

Figure S11: Simulated angle-dispersive mode deposition maps as a function of k_x in the positively displaced nanorod metasurface with the dye-doped film thickness of 380 nm.

References

(1) Liang, M.; Andreani, L. C.; Berghuis, A. M.; Pura, J. L.; Murai, S.; Dong, H.; Sánchez-Gil, J. A.; Gómez Rivas, J. Tailoring directional chiral emission from molecules coupled to extrinsic chiral quasi-bound states in the continuum. *Photonics Res.* **2024**, *12*, 2462–2473.
